# The Interplay Between Innate Lymphoid Cells and the Tumor Microenvironment

**DOI:** 10.3389/fimmu.2019.02895

**Published:** 2019-12-13

**Authors:** Laura Ducimetière, Marijne Vermeer, Sonia Tugues

**Affiliations:** Innate Lymphoid Cells and Cancer, Institute of Experimental Immunology, University of Zurich, Zurich, Switzerland

**Keywords:** innate lymphoid cells, tumor microenvironment, crosstalk, immune evasion, immune modulation

## Abstract

The multifaceted roles of Innate Lymphoid Cells (ILC) have been widely interrogated in tumor immunity. Whereas, Natural Killer (NK) cells possess undisputable tumor-suppressive properties across multiple types of cancer, the other ILC family members can either promote or inhibit tumor growth depending on the environmental conditions. The differential effects of ILCs on tumor outcome have been attributed to the high degree of heterogeneity and plasticity within the ILC family members. However, it is now becoming clear that ILCs responses are shaped by their dynamic crosstalk with the different components of the tumor microenvironment (TME). In this review, we will give insights into the molecular and cellular players of the ILCs-TME interactions and we will discuss how we can use this knowledge to successfully harness the activity of ILCs for anticancer therapies.

## Introduction

In the past years, Innate Lymphoid Cells (ILCs) have emerged as crucial players in cancer growth and therapy. The ILC family members are classified into five groups namely Natural Killer (NK) cells, group 1 ILCs (ILC1s), group 2 ILCs (ILC2s), group 3 ILCs (ILC3s), and lymphoid-tissue inducer cells (LTis) (1, 2). Initially described in the 1970s, NK cells are the founding members of the ILCs family (3). They develop in the bone marrow from common lymphoid progenitors and follow a sequential maturation and differentiation process, which is regulated by a variety of transcription factors (4). The T-box protein 21 (T-BET) and Eomesodermin (EOMES), for instance, undertake non-redundant roles in this process through stabilizing distinct NK cell subsets during maturation (5). Once in tissues, NK cells are potentially capable of eliminating infected or transformed cells via several mechanisms including degranulation, death receptor ligation, or the production of inflammatory cytokines (e.g., IFNγ, TNFα) (6). The latter feature is shared with another member of the family, the ILC1s, which are phenotypically very similar to NK cells (7, 8). However, the two lineages have been shown to diverge early in ontogeny and to differ in terms of cytotoxic machinery and tropic properties (9). Thus, ILC1s are typically defined as tissue resident since they are not found in the blood or lymphoid tissues, but rather in organs such as the gut, the liver, the salivary glands or the reproductive tract (9). In the liver, for example, T-bet dependent ILC1s have been shown to contribute to immune responses against haptens and viral antigens (10). In contrast, the intestinal ILC1 subset rather controls microbial pathogens and contributes to chronic inflammation (11).

ILC2s are characterized by their ability to produce Th2 cytokines (IL-4, IL-5, IL-9, or IL-13) and therefore contribute to type 2 inflammation promoting pathological responses associated to asthma or allergy, but also conferring protection against helminths (12). ILC2s require the transcription factor GATA-3 for their development (13). In the mucosal tissues, where they typically reside, ILC2s can be activated by epithelial-derived alarmins (IL-33, IL-25, or TLSP), whose contribution to ILC2 activation depends on the tissue type as well as on the nature and magnitude of the pathological insult (14). Thus, whereas IL-33 is believed to play a crucial role in the context of allergic airway inflammation, IL-25 is particularly relevant for the amplification of type 2 immune responses in the gut (15, 16).

ILC3s comprise a heterogenous and plastic population. They are divided into two subsets based on the expression of the natural cytotoxicity receptor (NCR) NKp46 in mice and Nkp44 in humans namely NCR^+^ILC3s and NCR^−^ILC3s (17). Both subsets require the transcription factor RORγt for their development and represent a major source of the cytokine IL-22 (18), which regulates interactions with the commensal flora and controls mucosal infections at barrier sites (19). ILC3s can also take proinflammatory roles through the release of IL-17 or IFNγ, contributing to the progression of psoriasis and colitis, respectively (20, 21). The production of abundant amounts of IL-17 and IL-22 is also a defining feature of LTis, which functionally resemble the population of NCR^−^ILC3s (22). LTis, however, are derived from a developmental pathway starting in the embryo (23), where they engage in the formation of lymphoid tissues through the production of lymphotoxins (24).

The properties of ILCs have been widely investigated in the context of tumorigenesis. Due to their high cytotoxic capacity, NK cells are particularly suitable to eliminate tumor cells. Indeed, several preclinical studies have revealed a central role for NK cells in tumor control, especially in metastatic disease (25–30). ILC2s and ILC3s can also modulate antitumor responses, but their role rather depends on the environmental cues they encounter in their resident tissue. Thus, whereas IL-12-stimulated NCR^+^ILC3s were found to control primary melanoma growth (31), the growth of this tumor type in the lungs is modulated by an IL-5-producing subset of ILC2s (32). This contrasts with the protumorigenic role of ILCs described for other tumor models. For example, IL-13-producing ILC2s promote tumor growth in leukemia and breast cancer (33, 34), and IL-22-producing ILC3s do likewise in the gut (35, 36). Finally, the recruitment of RORγt^+^ILC3s to tumors mediated by CCL21 was able to promote lymph node metastasis by modulating the local chemokine milieu in the TME (37).

With the growing interest in harnessing ILC responses for immunotherapeutic strategies against cancer, it is important to better understand the multifaceted roles of ILCs in tumor development. Here, we will first discuss how ILCs migrate and expand in the tumor site. Further, we will review current knowledge on how ILCs communicate with the environment, including the interactions they establish with the tumor cells and the different components of the TME. Finally, we will discuss whether these interactions are beneficial or deleterious to tumor growth and invasion.

## ILCs in the Tumor Site: Migration vs. Local Expansion

Parabiosis studies have shown that NK cells are a highly mobile subset that constantly circulate throughout the bloodstream and the lymphatic system, whereas the rest of the ILC family members are defined as tissue resident cells (38–40). Confirming the maintenance by local self-renewal within tissues, only very small numbers of ILCs can be found in healthy human and mouse circulation (41, 42). It has been shown that some ILC progenitors express the integrin α4β7 and the chemokine receptors CCR7, CCR9, or CXCR6, which may enable them to migrate to peripheral and lymphoid tissues (42–44). Also mature ILCs express several tissue homing receptors such as CXCR6, which promotes the accumulation of ILC3s in the gut (45) and provides survival signals to maintain ILC1s within the hepatic niche (46). Other markers of tissue residency for ILCs are the integrin CD49a and the early activation marker CD69, which are upregulated during ILC activation (47–49).

Within the TME, NK cells represent by far the most abundant innate lymphocyte subset identified (48). However, despite correlating with a better prognosis, NK cell homing is highly inefficient in most solid tumors (50, 51). There are a few exceptions including clear cell renal carcinoma, which harbors unusually high numbers of intratumoral NK cells (52, 53). The mechanisms leading to NK cell paucity in the TME are not well-studied, but what it is by now clear is that the majority of NK cells infiltrating tumor tissue belong to the mouse CD27^high^ and the human CD56^bright^ subsets, which are recruited to the tumor in a CXCR3-dependent manner (54, 55). Even though the immature CD56^bright^ NK cell population has been traditionally considered as a “cytokine producer,” whether it can control tumors as efficiently as the mature CD56^dim^ population is still a matter of debate. Due to their high motility, NK cells can also be recruited in strategic locations in order to prevent further cancer spread. As such, highly cytotoxic populations of NK cells from both CD56^dim^ and CD56^bright^ subsets have been found in tumor-draining lymph nodes of melanoma patients (56, 57). On the other hand, immunosuppressive mediators such as TGFβ might favor the retention of NK cells in the bone marrow through the upregulation of CXCR4 and the downregulation of CX3CR1 (58).

Despite their residency properties, a few ILCs have been reported to circulate in human blood. Thus, increased frequencies of ILC1s and ILC2s were found in patients with colorectal cancer (59) and with gastric cancer, respectively (60, 61). RORγt^+^ILC3s were also reported to migrate via the bloodstream toward the tumor site in response to CCL21 in mouse models of breast cancer (37) and in melanoma (62). Within the TME, ILC subsets other than NK cells are only found at extremely scarce numbers. In human lung cancer, a NCR^+^ population of ILC3s was found to accumulate at the edge of lymphoid structures in the tumor tissue (37, 62, 63). An enrichment of ILCs in tumors compared to healthy tissue has also been observed for ILC1s in gastrointestinal tumors (49) or ILC2s in gastric, breast and prostate cancer (34, 49, 60). Despite the presence of ILCs in the above-mentioned types of cancer, whether they contribute to the underlying pathology in humans is still a matter of debate. Also, whether the enrichment of ILCs results from newly recruited cells or from local *in situ* proliferation has not been thoroughly investigated. The latter phenomenon was however observed for ILC2s in IL-33-treated breast cancer (33), and for ILC1s in mouse mammary pre-cancerous lesions (64).

## The Bidirectional Crosstalk Between ILCs and Tumor Cells: Recognition vs. Immune Evasion

From all the ILC family members, NK cells show the highest cytolytic activity, while the primary role of other ILCs is to produce cytokines in response to different stimuli. In order to eliminate transformed cells, NK cells are equipped with a plethora of activating and inhibitory receptors, which need to be tightly regulated to determine whether a target cell will be killed or spared (65). Once activated, NK cells eliminate target cells via death receptors pathways (e.g., Fas/FasL) or through the release of cytotoxic granules at the immunological synapse (66). The usage of these two cytotoxic pathways appears to be tightly regulated. As such, whereas NK cells use the fast granule-mediated pathways for their first killing events, they switch to death receptors-mediated killing during the last encounters with the tumor cells (67). Despite possessing such an efficient cytotoxic machinery, NK cells from tumor-bearing mice or cancer patients are often functionally impaired and display low amounts of effector molecules such as granzyme B, IFNγ, or FasL (68). This is mostly due to the signals these cells receive from the TME, and especially from the surrounding tumor cells.

Within the TME, tumor cells are constantly exposed to stress conditions, which induce the upregulation of ligands for NK cell activating receptors (69). Although *a priori* this would favor NK cell-mediated immune surveillance, cancer cells have developed several mechanisms that allow them to evade immune recognition. Among those, we highlight the dysregulation of ligands that bind NKG2D, a major NK cell activating receptor critical for antitumor immunity (70). A commonly proposed mechanism for evading NK cell surveillance has to do with the shedding of the NKG2D ligands MICA and MICB from the cell membrane, leading to soluble forms that promote the internalization and posterior degradation of the receptor (71–73). This was however challenged in a study performed in murine tumor models, which reported that the soluble high affinity NKG2D ligand MULT-1 actually caused NK cell activation and tumor rejection (74). Irrespective of whether NKG2D ligands are soluble or membrane-bound, what is clear by now is that it is their chronic engagement which causes the desensitization of the NK cell receptor as well as related signaling pathways (75). Moreover, although tumor cells represent the main source of ligands for activating receptors, the induction of NKG2D ligands on myeloid cells and endothelial cells has also been shown to contribute to impaired NK antitumor responses (76, 77). Finally, other ILC family members such as intestinal ILC1s and ILC3s can also express NKG2D on the cell surface (78). Whether this receptor is able to modulate the activity of these cells in the TME is however not known.

Besides desensitizing NKG2D, tumor cells use additional mechanisms to evade NK cell surveillance including the secretion of immunosuppressive molecules such as TGFβ, IL-10, prostaglandin E2 (PGE2) or indoleamine 2,3-dioxygense (IDO) (79, 80). The production of these factors is not restricted to cancer cells, and a variety of cell types populating the TME can also contribute to the immunosuppressive pool leading to impaired NK cell function. Nevertheless, TGFβ and PGE2 are able to shape NK cell activity directly via the inhibition of activating receptors (79–81), or indirectly through the recruitment of immunosuppressive cells types such as myeloid-derived suppressor cells (MDSCs) or regulatory T cells (Tregs) (82, 83).

ILCs have a remarkable plasticity allowing them to acquire features of another ILC population in order to adapt to changes in the tissue microenvironment. In tumors, ILC plasticity was suggested as a mechanism by Gao et al., who reported a TGFβ-dependent conversion of NK cells into “ILC1-like” cells in a mouse model of chemically induced sarcoma (84). This conversion, which is characterized by the upregulation of the integrin CD49a and the downregulation of Eomes, appears to be detrimental for tumor control (84). A similar CD49a^high^ ILC1-derived subset with a tissue-residency phenotype was however found to exert cytotoxicity in oncogene-induced murine tumor models (64). Given the overlapping phenotypes between NK cells and ILC1s (85), it is difficult to postulate whether one subset really converts into the other or if cells rather evolve on a continuum. A complete transition seems unlikely, since ILC1s and NK cells lineages are believed to separate early during the differentiation process (78, 86).

The dependence of NK cell into ILC1 conversion on TGFβ supports increasing evidence that this cytokine does not only induce NK cell dysfunction, but also plays a crucial role in regulating ILC plasticity. Interestingly, TGFβ-imprinting is essential for the differentiation of the ILC1s residing in murine salivary glands via suppression of Eomes and the upregulation of CD49a (87). In humans, TGFβ was also described to enable the transition between mature CD16^+^ peripheral blood NK cells into a CD16^−^CD9^+^ phenotype that resembles a population of decidual NK cells (88). But TGFβ is not the sole factor reported to induce ILC plasticity. In fact, the proinflammatory cytokine IL-12 was shown to induce the differentiation of ILC2s into IFNγ-producing ILC1s, a process that was reversed by IL-4 (89, 90). Further, IL-12 mediates the conversion of ILC3s into type 1-like ILCs in a variety of pathological conditions (31, 91–93). The so-called “ex-ILC3s” were found to display cytotoxic activity in humans (93) and to effectively suppress tumor growth in a mouse model of melanoma (31). In the context of intestinal inflammation, the ILC3 to ILC1 plasticity was reversible in the presence of IL-23 (92). Together, these results reinforce the notion that ILCs are highly plastic cells which fine-tune immune responses to adapt to the changing environment.

**Figure 1:**
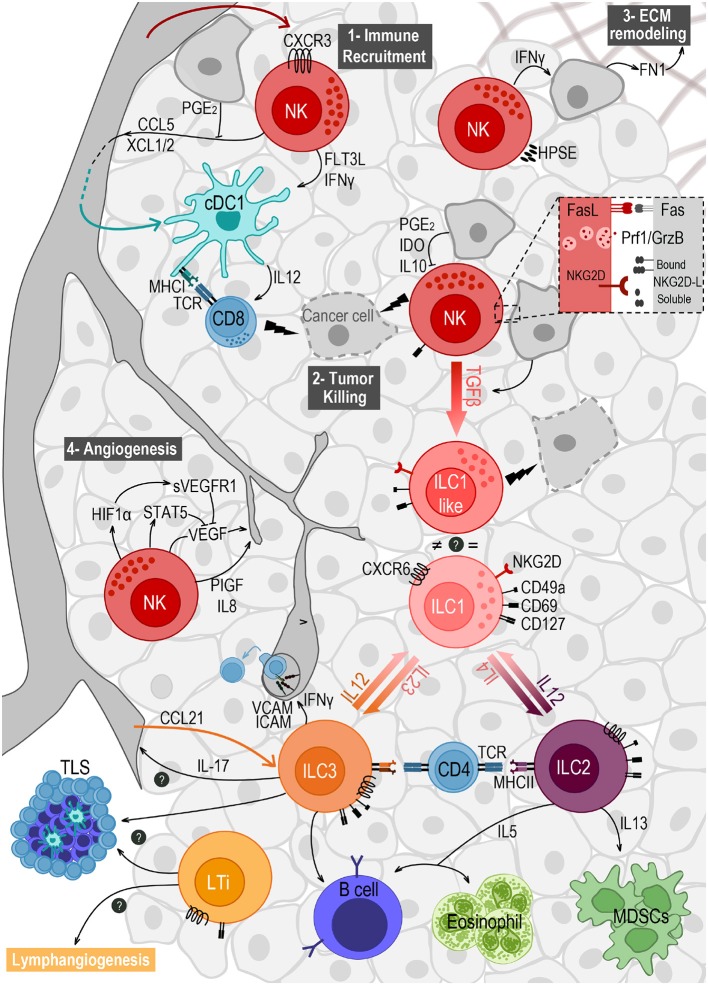
Crosstalk of ILCs and the different components of the tumor microenvironment. NK cells are the main ILC subset found in the TME, after migrating from the blood in a CXCR3-dependent way. They play important roles in (1) immune recruitment, (2) tumor cell killing, (3) extracellular matrix (ECM) remodeling, and (4) angiogenesis. (1) NK cells recruit cDC1s to the TME by secreting CCL5 and XCL1/2, and promote their survival and maturation through FLT3L and IFNγ. (2) NK cell mediated killing is mostly achieved by the engagement of death receptors (e.g., Fas/FasL) and by the release of cytotoxic granules containing perforin (Prf1) and granzymes (GrzB). This response can be triggered by the expression of stress markers on tumor cells, recognized by the receptor NKG2D. Ligands to this receptor (NKG2D-L) are membrane-bound but can also be shed and released in soluble form. (3) NK cells express the enzyme heparanase (HPSE) to degrade heparin sulfate proteoglycans, allowing them to migrate in the tumor tissue. NK cells' secretion of IFNγ induces the production of fibronectin 1 (FN1) by tumor cells, further remodeling the ECM. (4) NK cells modulate angiogenesis by releasing VEGF, PIGF and IL-8. VEGF secretion can be repressed by STAT5 or by soluble VEGF receptor (sVEGFR1) induced as a response to hypoxia. Tumor cells' secretion of TGFβ induces the conversion of NK cells into ILC1-like cells expressing CD49a and CD69, and exhibiting anti-tumor cytotoxic activity. Plasticity within the ILC family also includes the conversion from ILC2 to ILC1, and from ILC3 to ILC1, both induced by IL-12 and reversed by IL-4 and IL-23, respectively. ILC2s have a pro-tumorigenic role via secretion of IL-13 driving the expansion of myeloid-derived suppressor cells (MDSCs), and an anti-tumorigenic role through IL-5 mediated recruitment of eosinophils and activation of B cells. ILC3 can also stimulate antibody production by B cells, and can favor leukocyte recruitment to the TME when sensing IL-12, through IFNγ-mediated upregulation of ICAM-1 and VCAM-1. They may also play a role in angiogenesis through the production of IL-17. NCR^+^ILC3s and LTis accumulate in tertiary lymphoid structures (TLS) and may also promote lymphangiogenesis. Finally, both ILC2 and ILC3 express MHC class II and were able to prime CD4 T cells *in vitro*.

The wide number of events that take place in the TME to evade ILC surveillance have been summarized in Figure 1.

## ILCs as Modulators of Vascular Remodeling

Angiogenesis, the formation of new blood vessels from pre-existing ones, is needed to satisfy the increasing demand of oxygen and nutrients of the growing tumor. This process is supported by several immune cell types via the production of pro-angiogenic growth factors (94, 95). NK cells, for instance, were the first ILC subset reported to modulate tumor vascularization. Hence, a population of CD56^bright^CD16^−^ NK cells was shown to produce high amounts of the proangiogenic molecules VEGF, PlGF, or IL-8, leading to the formation of capillary-like structure in patients with NSCLC, melanoma, breast and colon carcinoma (96, 97). Interestingly, this population is reminiscent of a CD56^bright^CD16^−^ subset of decidual NK cells, which may be involved in the tissue remodeling process associated with angiogenesis during embryonic development (98).

NK cells are able to regulate the production of the proangiogenic factor VEGF through various mechanisms. Firstly, the expression of VEGF can be repressed by the transcription STAT-5, leading to inhibition of angiogenesis and tumor growth (99). Since STAT-5 is required for NK cell cytotoxicity, it was proposed that cytokines that signal through this transcription factor (e.g., IL-2 and IL-15) may regulate tumor growth by promoting the conversion from angiogenic to cytotoxic NK cells (99). NK cells can also regulate their own production of VEGF when adapting to hypoxia (100). Thus, the induction of HIF-1α on NK cells induces the upregulation of the soluble receptor VEGFR-1 (sVEGFR-1), which sequesters VEGF leading to the formation of more functional vessels that induce tumor growth (100). Further, ILCs can induce changes in the tumor vasculature through the modulation of adhesion molecules (101, 102). A tumor-suppressive subset of IL-12-driven NKp46^+^ ILC3s promoted leukocyte recruitment through the induction of the adhesion molecules ICAM and VCAM on the tumor vessels (102), similarly to what was observed by tumor-infiltrating NCR^+^ILC3s in NSCLC tissues (63). ILC3s producing IL-17 may also play a role in regulating the tumor vasculature. IL-17 induces blood vessel permeability in pulmonary endothelial cells, thus leading to metastatic growth (103). Further, IL-17 signals through stromal cells to induce a variety of proangiogenic factors (e.g., VEGF, TGFβ, or IL-8) (104, 105). Finally LTis may also play a role in promoting lymphatic vessel growth, which actively participates in metastatic tumor dissemination (106). LTis interact with Mesenchymal Stem Cells (MSCs), which produce pro-lymphoangiogenic factors such as VEGF-C (107). Although the LTi-MSC crosstalk has been proposed to mediate lymph node metastasis in breast cancer (37), the involvement of the lymphatic vasculature in this setting remains unknown at this time.

Not only the vasculature, but also the extracellular matrix (ECM) is modified during the course of cancer progression. The ECM is a complex network of proteoglycans and fibrous proteins that support the surrounding cells and provide molecular cues for cell migration and differentiation (108). During cancer progression, the deregulation of the ECM promotes invasion, angiogenesis and facilitates immune cell infiltration (109). It has been shown that NK cells can modulate the ECM through the secretion of fibronectin 1 (FN1), leading to structural changes in the primary tumor and decreased metastasis (110). In addition, NK cells can facilitate their own migration through the ECM thanks to the expression of heparanase, an enzyme known to degrade heparin sulfate proteoglycans (HSPGs) (111). This raises concerns about the use of EMC inhibitors to block tumor cell invasion, since it may be detrimental for a proper migration of NK cells and possibly other subsets of immune cells.

Taken together, these reports highlight the importance of ILCs in modulating the tumor vasculature and the remodeling of the ECM (Figure 1), which could be exploited for immunotherapeutic purposes. Further work will have to address specific contributions of the distinct ILC subsets to this process. For instance, whether and how ILC2-signature cytokines regulate the angiogenic process has yet to be studied.

## ILCs Interact with a Broad Spectrum of Immune Cells within the TME

ILCs establish continuous interactions with a wide variety of cells within the TME. As such, understanding the nature of this crosstalk is crucial to unleash the full potential of ILC responses against developing tumors. Defined NK cell interactions in the cancer context include the interplay with DCs, the main sentinels of the innate immune system (112, 113). DCs can support NK cell responses through the secretion of several proinflammatory cytokines (IL-12, IL-15, IL-18, and Type I Interferon) (114). NK cells can in turn trigger DC maturation via the production of IFNs and Tumor Necrosis Factor (TNF) (115, 116). Within the TME, NK cells promote the recruitment of cDC1s, the DC subset capable of priming tumor-specific CD8 T cells (117). This is mediated through the secretion of CCL5 and XCL1/2 by intratumoral NK cells, and antagonized by PGE2 produced by the tumor cells (113). Apart from promoting the recruitment of DCs, NK cells can also prime and ensure their expansion. Thus, NK cells activated by MHC Class I low tumor cells can prime DCs to produce IL-12 and to induce protective CD8 T cell responses (118). Further, they appear to be the main source of the cytokine fms-like tyrosine kinase 3 ligand (FLT3L), a survival factor for DCs (112, 119). In contrast, the use of less immunogenic tumor cells led to the inhibition of DC activation by NK cells, which was mediated by the (TNF)-related apoptosis-inducing ligand (TRAIL) (120). This controversy was not observed in the human disease, where a high expression of NK cell and cDC1 signatures correlated with better prognosis and response to immunotherapy in a wide array of cancers (112, 113).

NK cells are not the only ILC subset that is able to shape myeloid cell responses. ILC2s, for instance, have been shown to either limit anti-tumor responses by triggering the expansion of MDSCs via secretion of IL-13 (34), or to enhance anti-cancer immunity by cooperating with DCs or eosinophils in the lung in a IL-5-dependent manner (32, 121). Also the crosstalk between ILC1s, ILC3s and myeloid cells has been shown to promote chronic inflammation leading to tumor initiation. First, ILC1s and “ex-ILC3s” have been found to accumulate in chronically inflamed guts in response to myeloid-derived cytokines such as IL-12 or IL-15 (47, 91). In this scenario, these two ILC subsets secrete high amounts of IFNγ, which engages neutrophils and macrophages to cause tissue injury (47, 122). Further, group 3 ILCs are particularly responsive to IL-23, a key pathogenic inducer of chronic intestinal inflammation (123). IL-23, which is primarily produced by cells of the myeloid lineage, induce the production of GM-CSF, IL-17 or IL-22 by ILC3s (20, 124, 125). Whereas GM-CSF feeds back on the myeloid cells to promote tissue damage and colitis (20, 125), IL-17 and IL-22 limit inflammation by maintaining the integrity of the epithelial barrier (126, 127). This contrasts with the protumorigenic role that both IL-22 and IL-17 exert in colorectal cancer (105, 128), where they were shown to have pro-proliferative and pro-angiogenic functions, respectively (105, 128).

In humans, the levels of IL-17 appear to be upregulated in colorectal cancer patients, where they associate to poor prognosis (35, 129, 130). Further studies will be required to determine the contribution of ILC3s to the total pool of IL-17 or IL-22-producing cells. Nevertheless, blocking the ILC-myeloid axis in tumors of intestinal origin arises as a promising approach and may represent a promising anti-cancer therapeutic strategy.

ILCs may also directly modulate the quality of T cell responses without prior DC crosstalk. NK cell-secreted IFNγ, for example, was shown to promote Th1 polarization in the lymph nodes in mouse models of infection (131, 132). A close cooperation between NK cells and T cells has also been shown in established lung carcinoma, where the stimulation of NK cells induced the recruitment of highly active T cells, leading to a more efficient tumor control (133). Also the engagement of NK cells with the TNF superfamily member LIGHT was found to trigger CD8 T responses at the tumor site (134). ILC2s can also modulate adaptive immune responses. The IL-33/ST2 signaling pathway, which drive ILC2 activation, shape an immunosuppressive microenvironment during intestinal tumorigenesis dominated by regulatory T cells (Tregs) (135). A possible mechanisms by which ILC2s might shape the Treg phenotype is through production of AREG, an EGF-like growth factor that enhances regulatory T cell functions (136), or via the production of Arginase 1 (Arg1), which inhibits T cell activation (137).

Upon activation, both ILC2s and ILC3s were able to upregulate the expression of MHCII molecules (138–142). In the case of ILC3s, this was accompanied by high levels of costimulatory molecules and the capacity to process antigens, thus promoting *in vitro* CD4 T cell priming (141). Whether this priming can also take place in the tumor setting is currently now known. Interestingly enough, the population of NCR^+^ILC3s described by Carrega et al. in human tumors was found to be located at the edge of tertiary lymphoid structures, an ectopic hub for acquired immune responses (63). Not only T cells, but also B cell responses can be regulated by ILCs. ILC2s, for instance, can modulate B cell function and antibody production through the production of IL-5 and expression of CD40 ligands (143, 144). Also, a population of splenic RORγt^+^ ILC3s located in the marginal zone was shown to help B cells for antibody production (145).

Collectively, the above-mentioned studies demonstrate that ILCs are poised to interact with other immune cells within the TME, and thereby modulate both innate and adaptive immune responses against tumors (summarized in Figure 1).

## Concluding Remarks

Manipulating ILC responses has emerged as an attractive therapeutic strategy against cancer. In principle, NK cells are the best-suited members of the ILC family to exploit for therapeutic purposes, due to their indisputable cytotoxic properties. In the past years, however, other ILC subsets endowed with either pro- or anti-tumor activity have gained increasing attention as important modulators of the TME. Irrespective of the subset, ILC therapies face a major obstacle, namely the poor capacity to infiltrate and to survive in the hostile tumor bed (50, 51). To date, we still miss a complete picture of the mechanisms regulating ILC migration into tumors. This would be of high interest not only to enhance the number of endogenous ILCs that reach the tumor, but also to increase the efficacy of cell transfer therapies. The latter includes, for instance, the utilization of NK cells engineered to express chimeric antigen receptor (CAR) specific for tumor antigens, which arises as a safe off-the-shelf therapy against refractory malignancies (146).

Once ILCs reach the tumor site, they encounter a hostile microenvironment, which imposes several limitations to dampen the activity of ILCs. Although tumor cells are the key drivers of NK cell dysfunction, other immunosuppressive cells populating the TME can significantly contribute to this process. The TME is characterized by a high degree of intra- and inter-tumor heterogeneity, which challenges the identification of targetable factors aimed at restoring tumor surveillance by ILCs. Nevertheless, a better understanding of the mechanisms that ILCs utilize to communicate with the TME will be key to effectively manipulate these cells for site-specific anticancer therapies. This can be achieved using multi-omics approaches that allow for the integration of data from diverse platforms, including single-cell transcriptomics, cytometry by time-of-flight (CyTOF) or multiplexed tissue imaging. A detailed and personalized multi-omics profile of the TME will be crucial for the design of novel approaches for cancer immunotherapy in the era of precision medicine.

It is important to point out that most of the studies directed to investigate ILCs-TME interactions relies on the use of non-specific strategies to deplete ILC subsets (e.g., blocking antibodies against asialo GM1, CD25, or CD90.2). Although this is being gradually substituted by genetic tools that allow for selective ablation and mapping of ILCs (147), the high plasticity observed within the different ILC family members complicates our ability to track and target these cells in the TME. The matter is further complicated for ILC1s, due to the lack of ILC1-knockout mice or antibodies that specifically deplete this ILC population. This calls for caution when interpreting the effects of the ILC1 population on both physiological and pathological conditions.

Clearly, as we only now start to understand the complex biology of the ILC family members, it is time to study the power of these cells not only from the direct effects they exert on cancer cells, but also from their ability to communicate with the TME. This will provide valuable insights into how to effectively manipulate ILCs for immune-mediated anticancer therapies.

## Author Contributions

All authors participated in the intellectual conception of the review, drafts, and final approval of the manuscript.

### Conflict of Interest

The authors declare that the research was conducted in the absence of any commercial or financial relationships that could be construed as a potential conflict of interest.
